# What We Do Not Know about Fungal Cell Adhesion Molecules

**DOI:** 10.3390/jof4020059

**Published:** 2018-05-17

**Authors:** Peter N. Lipke

**Affiliations:** 1 Biology Department, Brooklyn College, City University of New York, Brooklyn, NY 11210, USA; plipke@brooklyn.cuny.edu; Tel.: +1-718-951-5000 (ext. 1949); 2 The Graduate Center, City University of New York, New York, NY 10016, USA

**Keywords:** adhesin, mannoprotein, genomics, fungal biofilm, cell wall, adhesion array

## Abstract

There has been extensive research on structure and function of fungal cell adhesion molecules, but the most of the work has been about adhesins in *Candida albicans* and *Saccharomyces cerevisiae.* These yeasts are members of a single ascomycete order, and adhesion molecules from the six other fungal phyla are only sparsely described in the literature. In these other phyla, most of the research is at the cellular level, rather than at the molecular level, so there has been little characterization of the adhesion molecules themselves. A catalog of known adhesins shows some common features: high Ser/Thr content, tandem repeats, *N*- and *O*-glycosylations, GPI anchors, dibasic sequence motifs, and potential amyloid-forming sequences. However, none of these features is universal. Known ligands include proteins and glycans on homologous cells and host cells. Existing and novel tools can exploit the availability of genome sequences to identify and characterize new fungal adhesins. These include bioinformatics tools and well-established yeast surface display models, which could be coupled with an adhesion substrate array. Thus, new knowledge could be exploited to answer key questions in fungal ecology, animal and plant pathogenesis, and roles of biofilms in infection and biomass turnover.

## 1. Introduction: The Breadth of the Problem

Adhesion is a first step in biofilm formation as well as in pathogenesis, and so adhesion underlies many consequences of fungal lifestyle, commensalism, and infection. Also, adhesion is a first step in saprophytic interactions, critical for elemental cycling in the biosphere. The role of fungal cell adhesion in pathogenesis of humans is well known, and there is much research in preventing fungal biofilms on epithelia, which can lead to infections. Biofilms on indwelling catheters are a major source of human morbidity and mortality. Fungal adhesion in plant pathogenesis is just as critical, but less studied. Among the million(s) of fungal species that stick to things, the problem is even broader: it is surprising to learn that fungi can adhere to and grow in fuel lines, degrading fuel and creating blockages that can cause engine failures [1,2,3]. There are also rock-bound fungi and lichens [4]. Clearly, the consequences of fungal adhesion are widespread, and knowledge of the adhesins themselves would allow us to rationally design interventions to inhibit harmful interactions and promote beneficial ones. Therefore, this review summarizes the current state of knowledge about fungal adhesins and finishes by pointing to some important needs.

### 1.1. Some Relevant Reviews

A review on adhesins from fungal pathogens of humans was published in 2013 [5], and a recent chapter by Epstein and Nicholson focuses on adhesion of plant pathogens and secreted fungal “glues” [6]. Another excellent and broad survey is now a decade old [7], as is a biochemically oriented review on adhesins from *C. albicans* and *S. cerevisiae* [8]. These resources, together with more recent data, give an idea of what is known, unknown, and what needs to be done.

### 1.2. Genomics and the Taxonomy of Fungal Cell Adhesion

**Figure 1:**
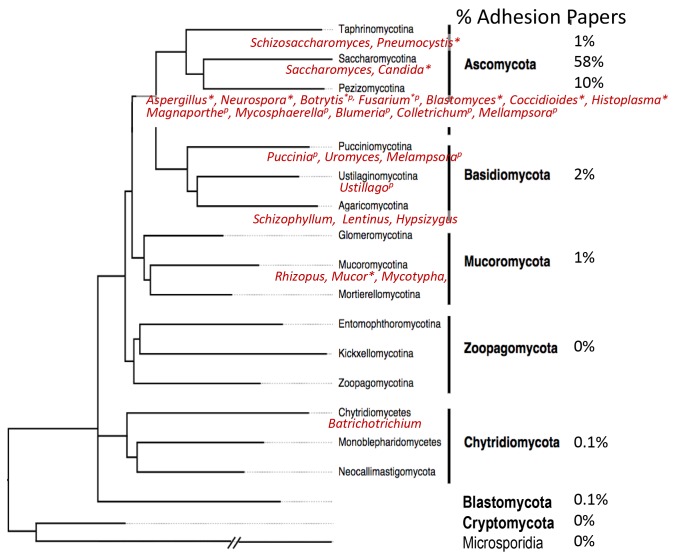
Fungal phylogenetic tree (adapted from [10]) showing the phyla (bold font) and subphyla. In red are the names of some genera whose cell adhesion has been studied. The genera marked * include common human pathogens, and genera marked *^p^* include top 10 plant pathogens. On the right is the fraction of papers attributable to each clade that include “adhesion” in the title.

Thanks to inexpensive genome sequencing and the 1000-fungal genome project, there is now a consensus on fungal phylogeny (Figure 1) [9,10]. There are seven phyla: (1) Ascomycota and (2) Basidiomycota (together these two phyla constitute the superphylum Dikarya), (3) Mucormycota, (4) Zoopagomycota, (5) Blastocladiomycota, (6) Chytridiomycota, and (7) Cryptomycota. The ‘phylum’ Zygomycota is now known to be polyphyletic, so its former members are assigned to other phyla on the basis of sequence similarities.

A February, 2018 expedition down into the Pubmed data-mine reveals biases in cell adhesion research. Of 1.5 × 10^6^ articles that mentioned “fungi”, about 1% included the term “cell adhesion”. Only 3800 articles (0.25%) had “adhesion” in the title. Searches by phylum gave few hits, e.g., only 474 for “ascomycete OR Ascomycota” and 25 for “basidiomycete OR basidiomycota”. Authors are more likely to state the genus and species, so data were mined by the name of the genus. 2200 articles (58% of those with “adhesion” in the title) were about *Candida* or *Saccharomyces*, ascomycetous yeasts in the order Saccharomycetales. 384 articles (10%) were about adhesion in the subphylum Pezizomycotina, the filamentous Ascomycota including *Aspergillus* and *Fusarium.* 52 articles (1%) were about the third Ascomycota subphylum Taphrinomycotina, which includes AIDS-associated opportunistic pathogen *Pneumocystis jirocvecii* (formerly *P. carinii*). Basidiomycota (which includes mushrooms as well as *Cryptococcus*) accounted for 103 articles (2%). Two other fungal phyla are represented by a single paper [11]. As expected, the sample is also highly biased towards human pathogens. Articles about the top 10 human pathogenic fungi (according to Wikipedia and [5,7]) outnumber the top 10 plant pathogens [6,12] by about 20:1. There were also three adhesion articles on the frog pathogen *Batrachochytrium* (phylum Chytridiomycota [11,13]) and none about *Pseudogymnoascus*, the filamentous Ascomycota that is the white-nose syndrome pathogen of bats. In sum, descriptions of adhesins are rare in phyla other than Ascomycota, and especially so in the five phyla that are not Dikarya. Thus, there is an opportunity for bioinformaticians to data-mine the fungal genomes for adhesin-related ORFs.

## 2. An Incomplete Catalog of Fungal Adhesins

### 2.1. Ascomycota

#### 2.1.1. Saccharomycotina

**Figure 2:**
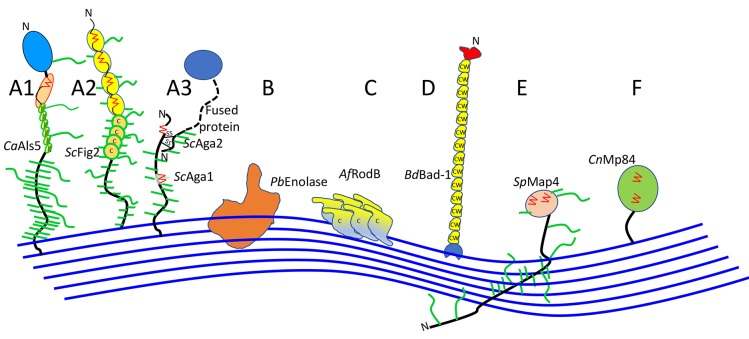
Cartoon models of some fungal adhesins, illustrating different domain arrangements and cell wall associations. A cell wall is shown as blue lines, representing glucan polymers. For each cartoon, abbreviations for the genus and species are in italics: *Ca*, *C. albicans*; *Sc*, *S. cerevisiae*; *Pb*, *Paracoccidioides braziliensis*; *Af*, *Aspergillus fumigatus*; *Blastomyces dermatitidis*; *Sp*, *Schizosaccharomyces pombe*, *Cn Cryptococcus neoformans.* The name of each adhesin is given in Roman font. Hydrophobic domains are filled in yellow. Potential amyloid-forming β-aggregation core sequences are shown as red zigzags; *O*-linked glycosylations are short green lines, *N*-glycans are longer green lines. C represents Cys-rich sequences in *Sc*Fig2 (**A2**) and *Af*RodB (**C**), and CW the Cys/Trp-rich domains in Bad-1 (**D**). Adhesins labeled (**A**) are covalently attached to the wall through modified GPI anchors, and (**F**) may be as well. The other sub-figure indices (**B** through **E**) show other cell wall attachment modes and are described in the text.

The vast majority of well-characterized fungal adhesins are from two genera in this subphylum, *Saccharomyces* and *Candida* (Figure 2A, Figure S1, and Table 1). These adhesins are often 600–2500-residues mannoproteins, covalently bound to cell wall glucan through modified GPI anchors. Many have discrete ligand binding domains (Figure 2A1,A3), but some do not (Figure 2A2). They can interact with homologous adhesions on fungi, bacteria, mammalian cells, or abiotic substrates. The binding mechanisms include ligand binding, hydrophobic effect, and amyloid-like protein–protein aggregation. These properties are summarized in Section 3, and extensively described in several review articles [5,6,7,14].

#### 2.1.2. Pezizomycotina

Pezizomycotina including *Aspergillus* and most of the “top ten” plant pathogens (Figure 1) [12]. Most articles in PubMed with title terms “*Aspergillus*” and “adhesion” are either about host factors for adhesion or reports of reduced adhesion after specific gene deletions. The deleted genes generally encode intracellular proteins that alter gene expression or cell surface structure, so there is little to help us deduce structure, identity, or function of putative adhesins.

##### Adhesins from Pezizomycotina

Fungal hydrophobins mediate binding to hydrophobic surfaces, including to plant hosts (Figure 2C and Figure S1) [15,16,17,18]. These are small Cys-rich proteins that self-assemble through amyloid-like interactions and coat the conidial surface in *Aspergillus* and other filamentous Ascomycota. Hydrophobins are a candidate for a protein expression tag because their self-aggregation provides a convenient method of concentration and purification of tagged macromolecules [16].

*Fusarium* and *Scedosporium* are Pezizomycotina human pathogens that appear occasionally in the adhesion literature. (Two *Fusarium* species also make the plant pathogen top-ten list [12]). A proteomics comparison of adherent and non-adherent strains of *F. oxysporum* has identified several potential GPI-anchored adhesins (Figure 2A) [19]. There are also identified candidates in *Scedosporium boydii* [20]. It is clear that such proteomic studies are potentially useful and the adhesins could be more certainly identified by funneling the ORF sequences through the FungalRV server described in Section 4 [21].

There is a clade of carnivorous Pezizomycotina that entrap and consume nematodes. They possess the adhesin Mad1, which has adhesin-like properties including secretion and GPI addition signals, β-aggregation-prone segments, and Cys-rich domains (Figure S1). When expressed in *S. cerevisiae*, Mad1 mediates binding to insect exoskeleton. Mad1 is under positive selection in the clade (i.e., evolutionary conservation), and so it must be important for survival within the clade [22,23].

The *Blastomyces WI-1* gene encodes the Bad-1 adhesin, which has a secretion signal, but no GPI addition signal (Figure 2D) [24]. It is highly repetitive, with 30–40 repeats of a 24-residue sequence with 2 Cys residues and 4 Trp residues, a highly unusual composition. Bad-1 is secreted into the media, then binds back to the cell surface through a C-terminal EGF-like chitin-binding domain. A BLAST Search shows that the chitin-binding domain is common in ORFs and putative adhesins from filamentous Ascomycota, so secretion and rebinding to chitin or glucan may be a common surface attachment motif among fungal adhesins.

Bad-1 has been recently structurally characterized [25]. An N-terminal HHPK sequence binds heparin with low affinity [26]. Mammalian protein disulfide isomerase (PDI) facilitates a major conformational shift by reducing and/or shuffling the native disulfides in each repeat. Tryptophan and basic residues then form ladders of stacked π-bonds that constitute additional heparin binding sites. The resulting activated molecule shows heparin binding at a large number of binding sites, and thus great avidity. This binding inhibits T-cell activation and contributes to immune escape in pathogenesis [26].

##### Genomics Approaches in Pezizomycotina

Two genomics-based studies illustrate approaches to adhesin identification in Pezizomycotina. One study compared transcripts in clinical isolates of *A. fumigatus* conidia highly adherent to airway epithelial cells vs. strains with low adherence [27]. Thirty-one transcripts showed robust correlations with adhesion and conidiation time. Of these, AFUA_4G09600 is increased >60-fold in the adherent strains, and it encodes a protein with multiple repeats, a predicted GPI anchor, and no predicted *N*-glycosylation sites (Figure 2A). Deletions of three of the candidate genes showed reduce adhesion to airway epithelium, although the reduction is not significant for *rodB.* Adhesion is restored by complementation of the deleted genes.

Another functional genomics approach is based on surface expression of putative adhesins in non-flocculent *S. cerevisiae* [28]. *A. nidulans* (*An*) and *A. fumigatus* (*Af*) ORFs with signal sequences and GPI addition sequences are expressed from vectors in a non-adhesive strain of *S. cerevisiae*. Of these, *AnMnpA*, *AnYpsA*, *AfMP1*, and *AfMP2* increase binding to fibronectin-coated beads and surfaces. The same study reports a screen of a cDNA library from *A. nidulans*, with selection for increased *S. cerevisiae* binding to fibronectin-coated beads. Among 21 candidate adhesins are *MnpA* and the glycolytic enzyme, fructose bisphosphate aldolase. Most of the other candidates are intracellular proteins involved in transcription, translation, and wall synthesis. The products of these genes probably affect adhesion by altering the surface of the yeast to make it adhere non-specifically, or by affecting expression of unidentified surface adhesins.

#### 2.1.3. Taphrinomycotina

Taphrinomycotina are the third branch of the Ascomycota tree (Figure 1), and among these, adhesins have been characterized in the fission yeast *Schizosaccharomyces* and the AIDS-related opportunistic pathogen, *Pneumocystis*. *S. pombe* Gsf2 is a galactose-specific lectin with a secretion signal and a GPI addition signal (Figure 2A1). Like Flo11 from *Saccharomyces cerevisiae* (*Sc*), it mediates agar invasion and formation of lentiform colonies within the agar [29,30].

From *Schizosaccharomyces pombe* and *S. japonica*, Linder and Gustafson identified a family of proteins with Ser/Thr-rich repeats and secretion signals, but none have GPI addition signals [31]. Instead, there are often recognizable lectin-like domains and/or a *Schizosaccharomyces*-specific domain called DIPSY, located at the C-terminal. The sexual flocculins Map4 and Mam3 are among the identified ORFs (Figure 2E). A subsequent characterization of Map4 shows that it cannot be released from walls with glucanases or reduction of disulfides but is solubilized with hot SDS or by 25 mM NaOH at 37 °C [32]. These characteristics are consistent with a non-covalent association with the wall, or perhaps esterification to glucan hydroxyl groups [33]. Another possibility is that the proteins are wall-bound through β-aggregation-prone sequences (17 sequences in Gsf2, 7 in Map4, and 9 in Mam3), which are prevalent in these adhesins. The Cys residues and C-terminal DIPSY domain are required for aggregation activity. The C-terminal DIPSY region necessary for cell–cell interaction is exposed at the outer surface of the wall.

*Pneumocystis* adhesins include *P. jirocvecii* Int1, an RGD-containing protein without a signal sequence, and a C-terminus region similar to the Bud4 GTP-binding protein [34]. Nonetheless, it appears to be expressed on the surface of cells, including when exogenously expressed in *S. cerevisiae.* Int1 expression in *P. jirocvecii* or *S. cerevisiae* mediates Ca^2+^ dependent binding to fibronectin. Therefore, this protein must be unconventionally secreted into the wall, like the glycolytic enzymes and heat shock proteins in the Saccharomycotina yeasts. *Pneumocystis MSG* (major surface glycoprotein) family includes about 80 ORFS, each of which can encode a predicted GPI-anchored protein. Only a single version is expressed at any given time, but the expressed versions change with immunological pressure in the host. Msg proteins are highly Cys-rich, and not prone to β-aggregation. Expression on cell walls increases adhesion to alveolar epithelial cells [35].

### 2.2. Basidiomycota

Relatively few adhesins are known from Basidiomycota. The best studied are those of the encapsulated yeast *Cryptococcus* (alternate name *Filobasidiella*) *neoformans* (alternate species name or variety *gatti).* A recent review discusses *C. neoformans* adhesion to A549 epithelial cells [36]. The capsular polysaccharide glucuronoxylomannan has been reported to have both adhesive and anti-adhesive properties. A more conventional adhesin is the mannoprotein MP84, which has a 411-residue ORF with secretion and GPI addition signals as well as *N*- and *O*-glycosylation sequences (Figure 2F) [36]. Soluble recombinant MP84 produced in *Pichia pastoris* binds to A549 lung epithelial cells and inhibits binding of a *C. neoformans* acapsular mutant to the same cells. These results are consistent with the idea that there is specific and saturable binding to an unknown ligand on A549 cells, and pretreating the cells masks all the ligand sites.

Another potential cryptococcal adhesin is CFL-1 [37,38], a 309-residue Cys-rich ORF with a secretion signal, an EGF domain, and a C-terminal CAAX motif (which can be a signal for isoprenylation [38]). The protein is secreted into the medium and is necessary for intercellular signaling leading to arial filamentation in mating. Its role in adhesion may be in signaling and induction of hyphal adhesins, rather than being an adhesin itself.

### 2.3. Other Fungal Phyla

The review by Epstein and Nicholson shows some of the diversity in other fungal clades and the Oomycota, which are in the Stramenopile kingdom, but show convergent characters with the fungi [6]. Most of the fungal adhesins are from Ascomycota or Basidiomycota. There is also a unique paper that describes screening fungal genomes for homologs of the GPCR 7-transmembrane receptors ([11]; this paper was the sole paper that included several phyla outside of the Dikarya in the Pubmed search for “adhesion[TITLE] AND fung*”. The paper includes a class of GPCR receptors with long extracellular domains that are known to mediate cell adhesion in mammalian cells. There are 30 novel fungal proteins of this class in Ascomycota, and 2 in *Allomyces* (Blastocladiomycota). However, none of the fungal homologs has a long enough extracellular domain to be exposed on the wall exterior surface, and therefore these ORFs are not expected to be adhesins. Nevertheless, this approach of looking for homologs of conserved protein classes can in theory yield new candidates for fungal adhesins.

## 3. Structural Characteristics of Known Fungal Cell Adhesins

### 3.1. “Typical” Adhesins

GPI-anchored adhesins in *S. cerevisiae* and *C. albicans* are the best-characterized fungal adhesion molecules: they are generally large mannoproteins (600–2500 amino acids and similar or greater numbers of sugars; Figure 2A and Figure S1) [8]. Each is synthesized with an N-terminal signal secretion signal, then many have a well-folded N-terminal domain that binds specific peptide or glycan ligands (Figure 2A1). There are Ser/Thr-rich tandem repeats, a long Ser/Thr-rich glycosylated stalk, and a peptide signal sequence that specifies addition of a glycosyl phosphatydylinositol (GPI) C-terminal glycolipid. The GPI anchor is added in the ER, then the glycan is cleaved at the cell surface, and the adhesin becomes crosslinked to cell wall β1, 6 glucan [8,14,39,40]. Homologs of known GPI-anchored adhesins have been identified in other species, and in a few cases they have been functionally assayed [41,42].

There are also *C. albicans* and *S. cerevisiae* cell surface GPI proteins such as *Ca*Hwp1, *Ca*Hwp2, *Ca*Pga22, and *Sc*Fig2 with similar length and amino acid composition, but without recognized N-terminal domains (Figure 2A2 and Aga1 in Figure 2A3). Many of these are expressed during biofilm formation, are necessary in model biofilm development, and some have demonstrated adhesion activity [43,44,45,46]. Sequence-based searches for homologs of these proteins are particularly prone to false results, because of the preponderance of low complexity regions and lack of recognizable structural domains [47].

### 3.2. Sequence Characteristics

Figure S1 shows the sequences of an arbitrarily chosen set of 24 adhesins, including several GPI-linked “typical” adhesins. Various sequence motifs are highlighted. The sequence features are summarized in Table 1, which shows that the vast majority have signal sequence (the two missing ones are probably due to database omissions). Twenty-one of the adhesins (88%) have regions with high TANGO β-aggregation potential [48], indicative of the ability to form amyloid-like interactions (see next section), and 18 (75%) have GPI addition signals. Among the domains, 16 (67%) have Cys-rich regions, and 18 (75%) have internal repeats. Most of the adhesins without internal repeats are short proteins. Fifteen adhesins have dibasic motifs, which are potential sites for proteolytic processing. Thus, a “typical” adhesin has a signal sequence, a GPI addition sequence, internal repeats, Cys-rich regions, *N*-glycosylation sites, and regions with high β-aggregation potential. In the few cases tested, *O*-glycosylations are extensive in the adhesins [8]. However, note that these “typical” characteristics are based primarily on adhesins from Ascomycota.

#### 3.2.1. N-Terminal Domains

Where fungal adhesins have recognized N-terminal domains, they are well-folded β-sheet domains. These are from several superfamilies: Ig-like invasins [49,50,51], FnIII domains [52], and the PAI-14 carbohydrate-binding lectins [53,54]. Each of these structures is represented in the PDB structural database.

#### 3.2.2. Tandem Repeats

Tandem repeats are common in adhesins and in other cell wall proteins [8,55,56]. There is structural information on the repeats in *B. dermatitidis* Bad-1, 24-residue disulfide-stabilized structures with a single α-helix [25]. The disulfides are rearranged to activate heparin-binding sites [26]. In another example, modeling of 36-residue repeats from *Ca*Als adhesins shows antiparallel β-sheet domains that have strong hydrophobic effect interactions. These domains unfold under force to further increase hydrophobic surface area [57]. This model can also apply to repeats in *Sc*Flo1. These 50-residue repeats have similar hydrophobicity and unfolding characteristics to those from *Ca*Als. In this case however, unfolding each repeat domain not only increases exposure of hydrophobic surfaces, but also exposes an amyloid core sequence [58,59,60,61]. Adhesion strength increases with the number of tandem repeats [62,63]. Thus, the best-characterized adhesin repeats contribute to hydrophobic effect binding to surfaces and other proteins, as well as amyloid-like aggregation [58,64,65].

#### 3.2.3. Cys-Rich Domains

Many adhesins contain clustered Cys residues in repeat domains (Figure S1). In addition to the Bad-1 heparin binding domains, examples including CX_4_C and WCPL motifs are present in the *S. cerevisiae* mating adhesins Aga1 and Fig 2, which interact with each other [66,67,68]. These Cys motifs in the first repeat in *Sc*Aga1 form disulfide bonds with *Sc*Aga2 subunit and are required for efficient mating [64,65]. Repeat sequences from *Ca*Hwp1 contain the same Cys motifs and can substitute for the *Sc*Aga1 sequence in chimeric proteins [66].

### 3.3. Cell Surface Attachments

Many families of well-researched adhesins are attached through GPI anchors modified by transglycosylation to cell wall glycans (Figure 2A). The model adhesins in Figure 2B and E have parts that appear to be enmeshed in the wall matrix, which would follow a model for invertase [69]. It is also possible, but not demonstrated, that adhesins are bound to glucan through ester bonds between carboxylated amino acids and glucan hydroxyl groups [31,33] however also see [32]. Another known attachment is through a chitin-binding domain (drawn as a blue C-terminal domain in Figure 2D; [25]).

### 3.4. Ligand Specificities

Ligand specificities are extremely diverse but are more often are tested by adhesion to target cells than by screening of defined molecules. Among ligand-binding activities, hydrophobic effect interactions are common and non-specific [5,8,57]. The best characterized of these interactions are in the fungal hydrophobins and *Ca*Eap1 [18,70,71]. In contrast, the wall-anchored adhesins with well-folded N-terminal domains usually bind specific ligands in a saturable manner. Such ligands include cell types, peptides, homologous or non-homologous proteins, and glycans expressed by homologous cells or hosts, summarized in Table 1 [5,8,72,73,74,75,76].

### 3.5. Amyloid-Like Interactions in Fungal Adhesins

Some well-characterized fungal adhesins interact through amyloid-like interactions that constitute long-lived bonds [58,77]. These adhesins use highly stable amyloid-like aggregation to cluster on the cell surface, increasing avidity. There is also evidence to support the idea that such bonds can form between cells to make very strong and long-lived cell–cell interactions [58,78]. It is now well established that *Ca*Als5 functions in this way, as well as the *S. cerevisiae* flocculins [58,60,61,77]. These interactions depend on presence of short (5–7 amino acid) sequences that can become exposed to solvent, and nucleate formation of β-aggregated amyloid-like plaques of adhesins on the cell surface. Cell–cell binding is inhibited or reversed by anti-amyloid compounds, and in the case of *Ca*Als5 the cell–cell binding is dependent on a specific sequence in the amyloid-forming region (Figure 2A). In *Ca*Als and *Sc*Flo adhesins, the amyloid core sequence is rich in three amino acids: Ile, Val, and Thr. The β-aggregate predictor TANGO identifies similar sequences in many fungal adhesins and potential adhesins (Table 1 and Figure S1) [48]. Speculatively, the formation of β-aggregated surface plaques may be a hallmark of fungal adhesins. Indeed, the *C. albicans* adhesins that do not have recognizable ligand binding domains are predicted to form amyloid-like interactions that could mediate cell–cell binding. Examples are the *C. albicans* Hwp family adhesins, Pga22, Pga59, and the GPI-anchored hydrophobin Eap1 [43,70,79,80]. Polyglutamine sequences in some adhesins (Table 1 and Figure S1) may also mediate adhesin self-association through protein–protein aggregation [81,82,83], as well as being substrates for host transglutaminases that crosslink surface-bound adhesins [81,82].

### 3.6. Cytoplasmic Proteins in Cell Adhesion

Glycolytic enzymes, heat shock proteins, and other cytoplasmic proteins are often localized within yeast cell walls (Figure 2B) [14,84]. These proteins are occasionally identified as adhesins or receptors for mammalian proteins [85,86,87]. Because these proteins have no secretion signal, the processes for secretion and wall association are unknown, as is whether the adhesion activity is fortuitous or important in pathogenesis or commensalism.

### 3.7. Are There Surface Molecules that Inhibit Adhesion?

Occasionally, an adhesin deletion leads to increased cellular adhesion of the deletant. These “anti-adhesins” show sequence features and motifs that are similar to the adhesins in Table 1. The increased adhesion in deletants could be due to increased expression of other adhesins, alteration of cell wall structure, or interference of the deleted adhesin with another adhesin. We have proposed the third model to explain how a deletion of the long adhesin *Sc*Fig2 increases activity of a shorter molecule α-agglutinin (*Sc*Sag1) by overtopping and masking [88]. Deletion of the GPI anchored cell wall protein Ywp1 in *C. albicans* increased adhesion of yeasts and hyphae [89,90]. Similarly, deletion of *Ca*Als5 increases adhesion of the yeast, perhaps by altering the cell surface amyloid-like aggregation in vivo [91]. One of the best described examples is *Ca*Pga22, which is an adhesin in its own right, but its overexpression decreases biofilm occupancy of overexpressing cells, and its deletion improves adhesion and biofilm participation [43]. Overexpression or deletion of Pga22 also changes cell wall structure.

### 3.8. Adhesion and Glycans

A few of the fungal adhesion references document roles of glycans in adhesion. In addition to their roles in fungal glues [6] and as ligands for lectin adhesins [72], they are also necessary for biofilm cohesion [92,93,94], fungal cell binding to surfaces [95], and aggregation of adhesins [96]. β-Glucans are an essential component of the matrix in *C. albicans* biofilms [45,97,98], and given their prevalence in fungal cell walls and the presence of molecular machinery for their synthesis and secretion, they may well be important in other fungal biofilms. Whether they mediate adhesion is still an open question. An *Aspergillus* polysaccharide containing galactosamine is essential for fungal binding to anionic surfaces [95]. The gene cluster responsible for synthesis of the polysaccharide is widespread in Pezizomycotina and is also found in a single Basidiomycota, *Trichosporon asahii*, a human commensal and opportunistic pathogen.

Two recent AFM-based papers describe indirect effects of glycans on adhesion. Host cell glycans are the subject of [96]. This paper shows that glycans of the *Candida* recognition receptor DC-SIGN, a mannose-specific lectin, are strengthened through glycan-mediated modulation of membrane rigidity, and subsequent stiffening of the cytoskeleton. On the fungal side, the α-mannan analog α1,4mannobiose is uniquely able to structure water to repel an AFM tip coated with the same saccharide [99]. Extra force is needed to promote interpenetration of the tip and substrate glycans and leads to a mannose-mannose association that also requires significant force to break. Thus, surface α-mannans may have the ability to resist approaching molecules and the ability to strengthen mannan–mannan interactions between cells. Therefore, the roles of glycans in cell adhesion include those of ligands for cell adhesion proteins, surface anchors, and modulators of fungal–host interactions [100] and fungal–fungal aggregation.

## 4. Bioinformatics and Discovery

The Thousand Fungal Genome project provides a large number of potential fungal adhesin sequences [9]. We will need new tools and approaches to make the datamining efficient and accurate.

### 4.1. Searching Databases for Adhesins

The known fungal adhesins are in several gene families, are highly diverse in sequence, and they evolve faster than other proteins [21,55,101]. Therefore, it is rare to find true homologs from different phyla. As an example, a BLAST search based on the *Ca*Als5 adhesin sequence using NCBI default parameters yielded 75 homologs, all from the *C. albicans*-related CUG clade, missing the *Sc*Sag1, a bona fide member of the *ALS* gene family. Also, the prevalence of low complexity regions rich in Ser, Thr, and Pro corrupts sequence searches, and calls proteins similar when they merely have similar amino acid composition [47,55]. The adhesins in Table 1 average 30% content for these three residues. Structure-based searches are more sensitive, but computationally more intensive, and there are few atomic-resolution structures on which to base a search [49,52,53,102].

One solution to the lack of query structures is FungalRV (http://fungalrv.igib.res.in/), a fungal adhesin predictor based on hidden Markov model (HMM) machine learning [21]. The input data is amino acid composition and frequency of dipeptides and tripeptides in a “learning set” of known fungal adhesins. The predictor was trained by comparing and contrasting compositional data for known adhesins to the amino acid composition and peptide frequencies of a large set of cytoplasmic proteins. The web server will consider a specific protein sequence, and score it for similarity to known adhesins, with a score of >0 being a positive predictor. Well-characterized GPI-anchored adhesins score at least +2. Standard cutoffs appear to give ~70% accuracy based on known adhesins, a number which probably reduces the number of ORFs to be tested from an entire genome to a few hundred genes. Thus, preliminary prediction of adhesins in a fungal genome is possible.

### 4.2. Is It Really an Adhesin? Some Approaches

**Figure 3:**
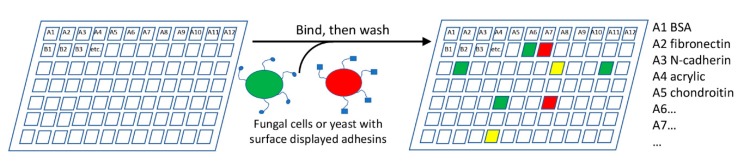
A fungal cell adhesion array would consist of a plate with different adhesion substrates printed or attached to the surface. Cells expressing adhesins would be incubated with the plate, and adherent cells scored for adherence after washing. The specific example shown shows two populations of cells labeled with red and green fluorescence, and the non-overlapping (red or green) or overlapping (yellow) adherence specificities.

The range of adhesion substrates that fungal cells can bind is unknown. A multiplexing approach to test adhesion to a variety of substrates would be highly useful (Figure 3). A chip or 96-well plate could be coated with a number of potential adhesion substrates, such as proteins, polysaccharides, or other biotic or abiotic materials. Fungi would be incubated with the plates, non-adherent cells washed off, and the plate inspected for adherent cells. Fluorescent labeling of the cells would allow easy automation, including comparing mutant and parental strains.

Mutants may show altered levels or specificities in adhesion, but deletions of upstream effectors also often reduce or increase adhesion, giving false positives [37,45,103]. A demonstration of adhesion activity of the gene product is preferable. In one approach, a fluorescently labeled protein (product of a specific candidate gene) could be screened on the array, as in the current use of lectin identification on glycan arrays [72,104]. Alternately, surface display of exogenous proteins in non-adherent laboratory strains of *S. cerevisiae* has been successfully used for over a decade [22,73,105]. GPI-anchored cell wall proteins are expressed from plasmids, and are glycosylated, secreted, and anchored in the cell wall. Proteins can also be expressed as fusions to *Sc*Sag1 [106]. A more frequently-used system is based on *Sc*Aga1-Aga2 surface expression, in which an exogenous protein is expressed as a fusion to Aga2, a 69-residue glycoprotein that is disulfide bonded to the GPI-anchored Aga1 (Figure 2A3) [107,108,109]. These fusion-based approaches have an advantage that they can be used in multiplexed experiments. Candidate genes would be bar-coded and fused to the display vector *Sc*Sag1 or *Sc*Aga2 sequence [109]. Surface expression would be followed by recovery of DNA from adherent cells and sequencing the fusion protein(s) would then reveal adhesin(s) mediating binding to each active substrate in the array. This technique can identify a range of active adhesins from a fungal genome. Gene expression studies would then catalog the conditions under which each adhesin is expressed.

## 5. What Do We Not Know?

The list of unknowns is long and can be divided into those that can be investigated with established procedures, and those that will require novel bioinformatic and experimental approaches.

### 5.1. Problems that Are Currently Soluble

#### 5.1.1. How Many Types of Fungal Adhesins Are There, and How Many in Each Fungus?

It is clear that a genomics-level approach can be a first step to learning whether more diverse fungal genomes contain ORFs homologous to known adhesins, or with characteristics like the known adhesins. Thus genomics-level screenings through FungalRV [21], or searches with recognized domains from fungal adhesins through ConSurf [110] will be informative but will need cross-validation. As an example, a ConSurf search through fungal sequences for homologs of the T-domain amyloid-forming region of *Ca*Als5 gave 1500 hits. This output needs to be screened for false positives caused by low complexity corruption [47,111], consilience with FungalRV [21], and correlated to distribution in the Fungal Tree of Life [9]. The identified ORFs can be catalogued for each of the characteristic features in Table 1. Clearly, a pipeline to analyze genome-level sequences would be highly useful. Genome-based identifications would be confirmed by screening deletion sets or overexpression libraries in each fungus on an adhesion array (Figure 3).

#### 5.1.2. How Widespread Are Amyloid-Based Clustering and Aggregation?

Sequence-based amyloid predictors are sensitive, but over-predict occurrence of functional amyloids, because many amyloid-prone sequences are buried inside stable domains or perhaps interfaces between domains [48,112,113]. Therefore, the genomic screen needs experimental confirmation. A potential easy approach is to assay for surface binding of thioflavin T or other amyloid dyes under shear stress in situ in the organism, or in yeast surface display [58,77,114,115]. It will also be possible to assess adhesion to an array in the presence and absence of inhibitory concentrations of anti-amyloid compounds (Figure 3).

#### 5.1.3. Are There More Roles for Cys-Rich Domains?

The remodeling of Cys-rich domains in Bad-1 lends experimental support to the idea that Cys-rich domains can be refolded at the cell surface. Such remodeling could either activate the adhesin (as in Bad-1) or facilitate covalent bond formation between cells. In the case of Bad-1, binding to heparin on the presence of reduced Cys residues. In other cases are disulfides present to prevent premature activation? Do disulfides mediate covalent integration into cell walls, like *Sc*Aga2? Why are some Cys residues and Cys-rich repeats essential and others not so?

For instance, *Ca*Pga13 is a GPI anchored surface protein with 5 repeats of a Cys rich sequence and multiple TANGO β-aggregation segments. Despite these primary structure features, *Ca*Pga13 protein is not known to be an adhesin, but it has not been assayed in the presence of reducing reagents. Its deletion increases cell surface hydrophobicity, cellular aggregation, and susceptibility to wall-stressing compounds. These phenotypes have been attributed to upregulation of compensating genes [116].

#### 5.1.4. Is There Covalent Bonding between Cells?

There has been little research into whether cell–cell adhesion can lead to covalent bond formation. A rare example is the idea that *Ca*Hwp1 is a substrate for mammalian transaminase [81]. Also, covalent crosslinks must occur in the cell wall as fungal cells fuse during mating and other cell fusion events, but do they occur in other cell–cell or cell–substrate interactions? As someone who has tried to remove mildew fungus from bathtub caulk, I am convinced that such bonding must exist. Among the potential intercellular bonds are amides, esters, and disulfides [33,117].

#### 5.1.5. What Are the Roles of Dibasic Sequences?

Are parts of adhesins shed, and if so, do they aggregate to form part of the matrix in biofilms? If the solubilized adhesin peptides have β-aggregating regions, they might self-associate to form crosslinked matrix material, as in bacterial biofilms [118,119].

### 5.2. Problems that Are Not So Easily Approached

- How widespread are adhesive glycans and what are their roles [6,93]?
- Structure and function of surface glues that stick fungi to hosts are open questions. An extreme example would be lichen-associated fungi,-associatedle wo which stick to rocks and have a mutualistic interaction with algae. How are glues related to extracellular matrix components in fungal and mixed fungal-bacterial biofilms [6,45]?
- What are the functions of “anti-adhesins”, proteins whose deletion leads to increased activity of other adhesins [91]?

### 5.3. Why Do We Need to Know More?

In these days of worldwide decreased funding for basic research, fungal cell adhesion is not a high priority for funding agencies. So what arguments are there?

#### 5.3.1. Evolution

Mammalian, bacterial, and fungal adhesins share domain structures, so fungal adhesins will continue to serve as models for mammalian adhesion molecules. This idea is supported by the success of yeast surface display in investigations of protein interactions (which after all is an example of adhesion) and ligand panning [73,105]. In addition, the roles of adhesins in biofilm formation appears to recapitulate their roles in organogenesis and development [120,121]. For evolutionary and developmental studies, the identification of specific roles for specific adhesins is a key to deeper understanding [46,66,74,122]. Also, the presence of apparent horizontal gene transfer affecting fungal adhesion in the presence of an adhesion gene complex in a single Basidiomycota genome argues for further investigations [95].

#### 5.3.2. Biofilms

Fungal adhesins are often identified as downstream effectors in developmental transcriptomics and proteomics studies of biofilms [45]. As biofilms develop, there is progression from one adhesin to another [45], and there is limited knowledge about the functional consequences of changes in adhesin expression. A related question is the role of outside-in signaling: How does a cell know it is “stuck down” to a substrate or to a neighboring cell? Quorum sensing is clearly important, but immobilized cells act differently from planktonic ones, and the adhesion itself must be a determinant. Are some adhesins parts of signal pathways, whereas others are not? How does the biofilm program change on different substrata, under different nutritional conditions, and under different biochemical stresses, or under different flow stresses? How does the presence of bacterial and other fungi in biofilms affect the biofilm program, and are the adhesins signalers, or merely downstream effectors? A few studies have begun to address this question [45,123,124]. The possibilities that adhesin-related developmental programs contribute to plant pathogenic biofilms and to saprophytic biofilms imply important roles for adhesins in human food supply and indeed the entire lifestyle of the planet.

#### 5.3.3. Pathogenesis

The questions listed in the Biofilms section above apply equally to pathogenesis. Is there a program of changes in adhesin as an infection progresses? Does that affect pathological biofilm production? If so, how is signaling altered by presence or absence of specific adhesins? Is the adhesin program altered by host immunity stresses? Adhesins can change the host immune response [25,45,125,126]. A recent paper documents differences in host response in different clinical isolates of *C. albicans* [127], and some of these differences may be due to differences in adhesin expression. If there is a developmental program for adhesins infections, is it relatively constant, or is it different in each host species? Or different in each host individual? Is it different in the presence of co-infection by bacteria or other fungi?

## 6. A Proposal

So, we return to the question of priorities. Clearly, genomics and big data are economical approaches to solving the problem of the ascomycetous and human-related biases. Data-mining for adhesin signatures can be a starting point, followed by deletion or overexpression studies with a substrate array. Positive identification as an adhesin would require demonstration of adhesive specificity by in vitro methods. In vitro biochemical and biophysical studies tell us a lot about mode of action but are not yet well-adapted to big data approaches. Three-dimensional structural studies are also still difficult for fungal adhesins, which are generally large and highly glycosylated. Therefore, an appropriate goal would be a catalog of adhesin expression during biofilm development and pathogenesis for diverse fungi (or at least diverse Ascomycota and *Cryptococcus*). Data-mining the results would reveal commonalities among programs, and subsequent experiments to alter the program(s) might demonstrate changes in the course of infections, commensal relationships, or environmental biofilms.

## Figures and Tables

**Table 1:** Summary of sequence-based features in fungal adhesins based on sequences in Figure S1. Each entry signifies presence of that feature in a specific adhesin. Columns show sequence length; presence of predicted secretion signal and GPI addition signals; presence of Cys-rich regions (CW denotes regions rich in Cys and Trp); presence of tandem repeats; dibasic sequences are KK, RR, KR, and RK; TANGO prediction of β-aggregation potential ≥10%; presence of ≥3 sequential Q residues; and known ligands (“Φ” represents hydrophobic effect binding); “?” denotes unknown or uncertain assignments. Organisms: *Ca*, *Candida albicans*; *Sc*, *Saccharomyces cerevisiae*; *Af*, *Aspergillus fumigatus*; *Po*, *Pleurotus ostreatus*; *Ao*, *Arthrobotrys oligospora*; *Bd*, *Blastomyces dermatitidis*; *Pj*, *Pneumocystis jirocvecii*; *Sp*, *Schizosaccharomyces pombe*; *Cn*, *Cryptococcus neoformans*.

	Figure 2 Model	Length (AA)	Signal	GPI	Cys-Rich Region	Tandem Repeats	Dibasic Sequence	TANGO >10%	*N*-glyco-sylation	QQQ	Ligands	Comments
**Ascomycetes**												
**Saccharomycotina**												
*Ca*Als2	A1	2362	X	X		X	1	11	22		oral epithelium	
*Ca*Als5	A1	1347	X	X		X	2	6	4		peptides, Φ, epithelial cells	very broad ligand specificity
*Ca*Eap1	A2	653	X	X	X	X	1	3	1		Φ	
*Ca*Hwp1	A2	634	X	X	X	X	1	3	3	X	Gln transaminase, buccal cells	
*Sc*Aga1	A3	725	X	X	X	X	-	5	-		-	SS bonded to Aga2
*Sc*Aga2	A3	87	X				-	1	-		*Sc* Sag1	SS bonded to Aga1; sexual adhesin
*Sc*Fig2	A1	2127	X	X	X	X	3	16	14		agar	secondary sexual adhesin
*Sc*Flo1	A1	1537	X	X	X	X	-	22	5		⍺-man	
*Sc*Flo11	A1	1367	X	X	X	X	2	4	3		*Sc* Flo11	homotypic binder
**Pezizomycotina**												
*Af*Hydrophobin	C	155	X	X	X	X	1	-	-		Φ	homopolymerizes
*Af*RodA	C	149	X	X			-	-	-		Φ	homopolymerizes
*Af*RodB	C	140	X	X	X		-	1	-		Φ	homopolymerizes
*Po* Veg Hyd 2	C	112	X	X	X		-	2	-		Φ	homopolymerizes
*Ao*Mad1	A2?	718	X	X	X	X	2	2	1		invertebrate exoskeleton	
*Bd*Bad1	D	1146	X		CW	X	1	2	-		heparin	
*Vd* Fas1-like	F	469	X	X				3	15		agar	
*Vd* Wsc1-like	A2?	468	X	X	X	X	4	2	-		agar	
**Taphrinomycotina**												
*Pj* Int1	?	1005			X	X	11	8	9		fibronectin	signal missing, PH/Bud4 domain
*Pj* Msg	A2?	1088		X	X	X	19	1	1		epithelial cells	signal missing
*Sp*Gsf2	A1	1563	X	X		X	-	13	23		galactose	
*Sp*Mam3	E	1082	X			X	-	10	9		*Sp* Map4?	sexual adhesin
*Sp*Map4	E	948	X		X	X	2	9	10		*Sp* Mam3?	sexual adhesin
**Basidiomycete**												
*Cn*Cfl1	?	309	X		CW	X	1	-	-	X		C-terminal CAAX sequence
*Cn*MP84	F	411	X	X			1	3	5		epithelial cells	chitin de-acetylase domain
**Entries in Table: 24**			**22**	**18**	**16**	**18**	**15**	**21**	**15**	**2**		

## Supplementary Materials

The following are available online at http://www.mdpi.com/2309-608X/4/2/59/s1. Figure S1: Sequences of some cell adhesion proteins and primary structure analyses.
