# Supplementary material for: What We Do Not Know about Fungal Cell Adhesion Molecules
Source: J Fungi (Basel). 2018 May 17;4(2):59. doi: 10.3390/jof4020059 (PMC6023273; doi:10.3390/jof4020059)
Supplement: Supplementary file 1 [file jof-04-00059-s001.pdf]

Figure S1. Sequences of some cell adhesion proteins and primary structure analyses.

Sequences were retrieved from NCBI in FASTA format. Repeat sequences are often visible as diagonal arrays of features. Potential signal sequences according to SIGNALP 4.1 (http://www.cbs.dtu.dk/services/SignalP/) are *italicized* and potential GPI signals (according to big-PI (http://mendel.imp.ac.at/gpi/fungi_server.html)) are *italicized in red*. Cys residues are highlighted in blue, dibasic sequences in magenta, N-glycosylation sequences in green Sequences to have β-aggregation potential ≥10% according to TANGO (http://tango.crg.es/) are highlighted in yellow.

Ascomycota

Saccharomycota:
Candida albicans
>sp|P0CU38.1|ALS2_CANAL RecName: Full=Agglutinin-like protein 2; AltName: Full=Adhesin 2; Flags: Precursor
*MLLQFLLLSLCVSVATA*KVITGVFNSFDSLTWTRAGNYAYKGPNRPTWNAVLGWSLDGTSANPGDTFTLN
MPCVFKFITDQTSVDLTADGVKYATCQFYSGEEFTTFSSLKCTVSNTLTSSIKALGTVTLPISFNVGGTG
SLVDLESSKCFKAGTNTVTFNDGDKKISIDVDFEKTNEDASGYFIASRLIPSINKASITYVAPQCANGYT
SGAMGFTIGSGDTTIDCSNVHVGITKGLNDWNFPVSSDSLSYNKTCSSTGISITYENVPAGYRPFFDVYT
SVSDQNRQLKYTNDYACVGSSLQSKPFNLRLRGYNNSEANSNGFVIVATTRTVTDSTTAVTTLPFNPSVD
KTKTIEILQPIPTTTITSYVGVTTSYSTKTAPIGETATVIVDVPYHTTTTVTSEWTGTITTTTTRTNPT
DSIDTVVVQVPSPNPTVXTTEYWSQSYATTTTVTAPPGGTDSVIIREPPNPTVTTTEYWSQSYATSSTVT
APPGGTDTVIIREPPNPTVTTTEYWSQSYATTTTVTAPPGGTDSVIIREPPNPTVTTTEYWSQSFATTTT
ITAPPGETDTVLIREPPNHTVTTTEYWSQSYVTTSTITAPPGGTDTVIIREPPNYTVTTTEYWSQSYATT
TTVTAPPGGTDTVIIREPPNPTVTTTEYWSQSYATTTTVTGPPGGTDTVIIREPPNPTVTTTEYWSQSYA
TTTTVTAPPGGTDTVIIREPPNPTVTTTEYWSQSYATTTTVTGPPGGTDTVIIREPPNPTVTTTEYWSQS
YATTTTVTAPPGGTATVIIREPPNPTVTTTEYWSQSYATTTTVTGPPGGTDTVIIREPPNPTVTTTEYWS
QSYATTTTVTAPPGGTATVIIREPPNYTVTTTEYWSQSYATTTTVTGPPGGTDTVIIREPPSPTVTTTEY
WSQSYATTTTVTAPPGGTATVIIREPPNPTVTTTEYWSQSYATTTTVTGPPGGTDTVIIREPPNPTVTTT
EYWSQSYATTTTVTAPPGGTATVIIREPPNYTVTTTEYWSQSYATTTTVTGPPGGTDTVIIREPPSPTVT
TTEYWSQSYATTTTVTAPPGGTATVIIREPPNPTVTTTEYWSQSYATTTTVTGPPGGTDTVIIREPPSPT
VTTTEYWSQSYATTTTVTAPPGGTATVIIREPPNYTVTTTEYWSQSYATTTTVTGPPGGTDTVIIREPPN
PTVTTTEYWSQSFATTTTVTAPPGGTDSVIIREPPNPTVTTTEYWSQSYATTTTVTAPPGGTDSVIIREP
PNPTVTTTEYWSQSFATTTTVTAPPGGTDSVIIREPPNPTVTTTEYWSQSYATTTTVTAPPGGTDSVIIR
EPPNPTVTTTEYWSQSYATTTTVTAPPGGTATVIIREPPNYTVTTTEYWSQSYATTTTVTAPPGGTATVI
IREPPNYTVTTTEYWSQSYATTTTITAPPGDTDTVIIREPPNYTVTTTEYWSQSFATTTTVTAPPGGTDS
VIIREPPNPTVTTTEYWSQSYATTTTVTAPPGGTATVIIREPPNYTVTTTEYWSQSYATTTTVTAPPGGT
ATVIIREPPNYTVTTTEYWSQSYATTTTITAPPGDTDTVIIREPPNYTVTTTEYWSQSYATTTTVTAPPG
GTDTVIIREPPNYTVTTTEYWSQSYATTTTVTAPPGGTATVIIREPPNYTVTTTEYWSQSYATTTTVTGP
PGSTDTVIIREPPNPTVTTTEYWSQSYATTTTVTAPPGGTATVIIREPPNYTVTTTEYWSQSYATTTTVT
APPGGTDTVIIREPPNYTVTTTEYWSQSYATTTTVTAPPGGTDTVIIREPPSPTVTTTEYWSQSYATTTT
VTAPPGGTATVIIREPPNYTVTTTEYWSESYATTTTVTGPPGGTDVILIREPPNPTVTTTEYWSESYATT
TTITAPPGATDSVRIREPPNYTVTTTEYWSQSYATTTTVTAPPGGTDSVIIREPPNPTVTTTEYWSQSYA
TTTTVTAPPGGTATVIIREPPNYTVTTTEYWSQSYATTTTVTAPPGGTDTVIIREPPSPTVTTTEYWSQS
YATTTTVTAPPGGTATVIIREPPSPTVTTTEYWSQSYATTTTVTAPPGGTATVIIREPPNYTVTTTEYWS
QSYATTTTVTGPPGGTDTVIIREPPNPTVTTTEYWSQSYATTLTITAPPGGTNSVIIRVHSSTNDESSES
TFSTLSVPSFSGSISVVSTVSRPHYVNSTVTHLPSSSSKPVDIPSSDVVTSTNDNSLTSLTGSENGKTSV
AISTTFCDDENGCQTSIPQGSVVRTTATTTATTTTIIGDNNGSGKSKSGELSSTGSVTTNTATPDVPSTK
VPSNPGAPGTGVPPPLAPSTETQTTNNVPGSPNIPATGTTDIIRESTTVSHTVTGNGNTGVPMNPNPVLT
TSTSLTGATNSATNPSHETSVNTGSGGSTNIVTPPSSATATVVIPGTDNGATTKGQDTAGGNSNGSTATT
NIQGGNNEPGNQPGTNTTGEPVGTTDTQSVESISQPTTLSQQTTSSLISTPLASTFDGS*GSIVQHSGWLY*
VLLTAISIFF

>sp|Q5A8T7.2|ALS5_CANAL RecName: Full=Agglutinin-like protein 5; AltName: Full=Adhesin 5; Flags: Precursor
*MIQQFTLLFLYLSFATA*KAITGIFNSIDSLTWSNAGNYAFKGPGYPTWNAVLGWSLDGTSANPGDTFILN
MPCVFKFTASQKSVDLTADGVKYATCQFYSGEEFTTFSSLKCTVNNNLRSSIKALGTVTLPIAFNVGGTG
SSVDLEDSKCFTAGTNTVTFNDGSKKLSIAVNFEKSTVDQSGYLTTSRFMPSLNKIATLYVAPQCENGYT
SGTMGFSTSYGDVAIDCSNVHIGISKGVNDWNHPVTSESFSYTKSCSSFGISITYQNVPAGYRPFIDAYI
SPSDNNQYQLSYKNDYTCVDDYWQHAPFTLKWTGYKNSDAGSNGIVIVATTRTVTDSTTAVTTLPFNPSV
DKTKTIEILQPIPTTTITTSYVGVTTSYSTKTAPIGETATVIVDVPYHTTTTVTSEWTGTITTTTTRTNP
TDSIDTVVVQVPLPNPTTTTTQFWSESFTSTTTITNSLKGTDSVIVREPHNPTVTTTEFWSESYATTETI
TNGPEGTDSVIVREPHNPTVTTTEFWSESYATTETVTNKPEGTDSVIIKEPHNPTVTTTEFWSESYATTE
TITTGPLGTDSIVIHDPLEESSSTTAIESSDSNISSSAQESSSSVEQSSSIVGLSSSSDIPLSSDMPSSS

STGLTSSESSTVSSYDSDSSSSSELSTFSSSESYSSSISDTTNFWDSSSSDLESTSITWSSSIDAQSSQS
VQSVSNSISTSQETTSSSGEESNTSVTDILVSSDASSILNSDISSYYPSSTISLSDDFPHTIAGEPDSRS
SSSIASTVEISSDLVSLTSDPTSSFDSSSSLNSDSSSSPFSDESDISASSSFSTLVAPSFSLSSSSSLSL
TYPHYVNSTTYHASESESSSVASPSMASESANDDTHTLSESTDTTSSIGTDSSTVTFCRRDNGDGCIVTG
MPSSSIDSEQTSDVTTTSSFVASSTPTSAEQSITDNPNIDSSQTSASSSTKSSVSVSDTVVNSISLSETS
TLSSDDSTSSDTSISSTTNSDTGNINAGSSHTSTASIKESSIQKTGVMLSSSYLSTKLSSTSDITTELIT
TELITTELTTIEDNEPNTFTSTPSSHSEIFSSDNSVLSKQVDRESTIKTSPTTDVTTVSSLSVHSTEAST
ATLGENSFSNVASTPSNIATSLRSTSSSSNHATESSGTVKSEASAEAIPSPPTSTDNRLSYSTEEAKGIT
YANSGSTNNLITESQVAAPTDSTSVLIENPVVTSTFDDNSSAAVDQPSKTKSIEESIMNPDSTNETNNGF
IATLSQAQVPNSLIHSESISTTMAKTTDASINGDSAASNSQPTTLIQQVATSSYNQPLITTYAGSSSATK
HPSWL*LKFISVALFFFL*

>AOW27933.1 Eap1p [Candida albicans SC5314]
*MKVSQILPLAGAISVASG*FWIPDFSNKQNSNSYPGQYKGKGGYQDDCGDDYKKGYKSKTYSKVKPITSTD
CTTPIQPTGTTTGYTKDVVESTSYTTDTAYTTTVITVTKCDGGSCSHTAVTTGVTIITVTTNDVITEYTT
YCPLTSTPATESTPATESTPATESTPATESTPATESTPATESTPCTTSTETTPATESTPATESTPATEST
PATESTPATESTPATESTPATESTPATESTPCTTSTETTPATESTASTETASSTPVESTVIVPSTTVITV
SSCYEDKCSVSSVTTGVVTISSEETIYTTYCPITSSITIPVPNTSTPAAPGTPVESQPVIPGTETTPAAP
GTPVESQPVIPGTETTPAAPGTPVESQPATTPVAPGTETTPAAPGTPVESQPATTPVAPGTETTPAAPGT
PVESQPVIPGTETTPAAPGTPVESQPATTPVAPGTETTPAAPGTPVESQPVIPGTETTPAAPGTPVESQP
ATTPVAPGTETTPAAPGTPVESQPVIPGTETTPAAPGTPGTEATPVTTQPVSVLSTSQVVTASGEFSTVT
AHSTSIVASCPEGGCVPEGQQTETSPSVPTNGPEVEASSSVLSIPVSSVTTSTIASSSETSVPPAQVSTF
EGS*GSALKKPYYGLAVAALVYFM*

>sp|P46593.5|HWP1_CANAL RecName: Full=Hyphal wall protein 1; AltName: Full=Cell elongation protein 2; Flags: Precursor
*MRLSTAQLIAIAYYMLSIGATVPQVDG*QGETEEALIQKRSYDYYQEPCDDYPQQQQQQEPCDYPQQQQQE
EPCDYPQQQPQEPCDYPQQPQEPCDYPQQPQEPCDYPQQPQEPCDNPPQPDVPCDNPPQPDVPCDNPPQP
DVPCDNPPQPDVPCDNPPQPDQPDDNPPIPNIPTDWIPNIPTDWIPDIPEKPTTPATTPNIPATTTTSES
SSSSSSSSSSTTPKTSASTTPESSVPATTPNTSVPTTSSESTTPATSPESSVPVTSGSSILATTSESSSA
PATTPNTSVPTTTTEAKSSSTPLTTTTEHDTTVVTVTSCSNSVCTESEVTTGVIVITSKDTIYTTYCPLT
ETTPVSTAPATETPTGTVSTSTEQSTTVITVTSCSESSCTESEVTTGVVVVTSEETVYTTFCPLTENTPG
TDSTPEASIPPMETIPAGSEPSMPAGETSPAVPKSDVPATESAPVPEMTPAGSQPSIPAGETSPAVPKSD
VSATESAPAPEMTPAGTETKPAAPKSSAPATEPSPVAPGTESAPAGPGASSSPKSSVLASETSPIAPGAE
TAPAGSSGAITIPESSAVVSTTEGAIPTTLESVPLMQPSANYSSVAPISTFEGAGNNMRLTF*GAAIIGIA*
AFLI

>CAA96325.1 AGA1 [Saccharomyces cerevisiae]
*MTLSFAHFTYLFTILLGLTNIALA*SDPETILVTITKTNDANGVVTTTVSPALVSTSTIVQAGTTTLYTTW
CPLTVSTSSAAEISPSISYATTLSRFSTLTLSTEVCSHEACPSSSTLPTTTLSVTSKFTSYICPTCHTTA
ISSLSEVGTTTVVSSSAIEPSSASIISPVTSTLSSTTSSNPTTTSLSSTSTSPSSTSTSPSSTSTSSSST
STSSSSTSTSSSSTSTSPSSTSTSSSLTSTSSSSTSTSQSSTSTSSSSTSTSPSSTSTSSSSTSTSPSSK
STSASSTSTSSYSTSTSPSLTSSSPTLASTSPSSTSISSTFTDSTSSLGSSIASSSTSVSLYSPSTPVYS
VPSTSSNVATPSMTSSTVETTVSSQSSSEYITKSSISTTIPSFSMSTYFTTVSGVTTMYTTWCPYSSESE
TSTLTSMHETVTTDATVCTHESCMPSQTTSLITSSIKMSTKNVATSVSTSTVESSYACSTCAETSHSYSS
VQTASSSSVTQQTTSTKSWVSSMTTSDEDFNKHATGKYHVTSSGTSTISTSVSEATSTSSIDSESQEQSS
HLLSTSVLSSSSLSATLSSDSTILLFSSVSSLSVEQSPVTTLQISSTSEILQPTSSTAIATISASTSSLS
ATSISTPSTSVESTIESSSLTPTVSSIFLSSSSAPSSLQTSVTTTEVSTTSISIQYQTSSMVTISQYMG*S*
GSQTRLPLGKLVFAIMAVACNVIFS

>CAA96733.1 AGA2 [Saccharomyces cerevisiae]
*MQLLRCFSIFSVIASVLA*QELTTICEQIPSPTLESTPYSLSTTTILANGKAMQGVFEYYKSVTFVSNCGS
HPSTTSKGSPINTQYVF

>KZV12934.1 FIG2 [Saccharomyces cerevisiae]
*MNSFASLGLIYSVVNLLTRVEA*QIVFYQNSSTSLPVSTLVSTSIADFHESSSTGEVQYSSSYSYVQPSID

SFTSSSFLTSFEAPTETSSSYAVSSSLITSDTFSSYSDIFDEETSSLISTSAASSEKASSTLSSTAQPHR
TSHSSSSFELPVTAPSSSSLPSSTSLTFTSVNPSQSWTSFNSEKSSALSSTIEFTSSEISGSTSPKSLES
LDTTGTITSSYSPSPSSKNSNQTSLLSPLEPLSSSSGDLILSSTIQATTNDQTSKTIPTLVDATSSLPPT
LRSSSMAPTSGSDSISHNFTSPPSKTSGNYDVLTSNSIDPSLFTTTSEYSSTQLSSLNQASKSETVNFTA
SIASTPFGTDSATSLIDPISSVGSTASSFVGISTANFSTQGNSNYVPESTASGSSQYQDWSSSSLPLSQT
TWVVINTTNTQGSVTSTTSPAYVSTATKTVDGVITEYVTWCPLTQTKSQAIGVSSSMSSVPQASSFSGSS
NSTALAASNNVPESTASGSSQYQDWSSSSLPLSQTTWVVINTTNTQGSVTSTTSPAYVSTATKTVDGVIT
EYVTWCPLTQTKSQAIGVSSSMSSVPQASSFSGSSNSTTLAASNNVPESTASGSSQYQDWSSSSLPLSQT
TWVVINTTNTQGSVTSTTSPAYVSTATKTVDGVITEYVTWCPLTQTKSQAIGVSSSMSSVPQASSFSGSS
NSTALAASNNVPESTASGSSQYQDWSSSSLPLSQTTWVVINTTNTQGSVTSTTSPAYVSTATKTVDGVIT
EYVTWCPLTQTKSQTIGVSSSMSSVPQASSFSGSSILSSNSSTLAASNNVPESTASGSSQYQDWSSSSLP
LSQTTWVVINTTNTQGSVTSTTSPAYVSTATKTVDGVITEYVTWCPLTQTKSQTIGVSSSMSSVPQASSF
SGSSILSSNSSTLAASNNVPESTASGSSQYQDWSSSSLPLSQTTWVVINTTNTQGSVTSTTSPAYVSTAT
KTVDGVITEYVTWCPLTQTKSQTIGISSSTISATETSKPSSILTLGISTLQLSDATFKGTETINTHLMTE
STSITEPTYFSGTSDSFYLSTSEVNLASSLSSYPNFSSSEGSTATITNSTVTFGSTSKYPSTSVSNPTEA
SQHVSSSVNSLTDFTSNSTETIAVISNIHKTSSNKDYSLTTTQLKTSGMQTLVLSTVTTTVNGAATEYTT
WCPATSIAYTTSISYKTLVLTTEVCSHSECTPTVITSVTATSSTIPLSSTSSSTVLSSTVSEGTKNPAAS
EVTTNTQVSATSEATSTSTSVTATASESSTTSQVSATSEATSSSTQVSATSVTATASESSTTSQVSATSE
ATSTSTSVTATASESSTTSQVSATSVTATSSESSTTSQVSTASETISTLGTQNFTTTGSLLFPALSTEMI
NTTVVSRKTLIISTEVCSHSKCVPTVITEVVTSKGTPSNGHSSQTLQTEAVEVTLSSHQTVTMSTEVCSN
SICTPTVITSVQMRSTPSPYLTSSTSSSSLVSTKKSSLEASSEMSTFSVSTQSLPLAFTSSEKRSTTSVS
QLSSTVLTNTIMPSSSNVISTNEKPSSTTSPYNFSSGYSLSSSSTPSQYSLSTATTTINGIKTVYTTWCP
LAEKSTVAASSQSSRSVDRFVSSSKPSSSLSQTSIQYTLSTATTTISGLKTVYTTWCPLTSKSTLGATTQ
TSSTAKVRITSASSATSTSISLSTSTESESSSGYLSKGICSGTECTQDVPTQSSSPASTLAYSTSVSTSS
SSFSTTTASTLTSTHTSVPLLPSSSSISASSPSSTSMLSTSLPSPAFTSSTLPTATAVSSSTFIASSLPL
SSKSSLSLSPVSSSILMSQFSSSSSSSSSSSSSSSSSSSSSSSSSSSSSSSSSSLASSPSLSISPTVDTV
SVLQPTTSIATLTCTDSQCQQEVSTICNGSNCDDVTSTATTPPSTVTDTMTCTGSECQKTTSSSCDGYSC
KVSETHKSSATISACSGEGCQASATSELNSQYVTMTSVITPSAITTTSVEVHSTESTISITTVKPVTYTS
SDTNGELITITSSSQTVIPSVTTIITRTKVAITSTPKPTTTTYVEQRLSSSGIATSFVAAASSTWITTPI
VSTYAG*SASKFLCSKFFMIMVMVINFI*

>sp|P32768.4|FLO1_YEAST RecName: Full=Flocculation protein FLO1;
Short=Flocculin-1; Flags: Precursor
*MTMPHRYMFLAVFTLLALT*SVASGATEACLPAGQRKSGMNINFYQYSLKDSSTYSNAAYMAYGYASKTKL
GSVGGQTDISIDYNIPCVSSSGTFPCPQEDSYGNWGCKGMGACSNSQGIAYWSTDLFGFYTTPTNVTLEM
TGYFLPPQTGSYTFKFATVDDSAILSVGGATAFNCCAQQQPPITSTNFTIDGIKPWGGSLPPNIEGTVYM
YAGYYYPMKVVYSNAVSWGTLPISVTLPDGTTVSDDFEGYVYSFDDDLSQSNCTVPDPSNYAVSTTTTTT
EPWTGTFTSTSTEMTTVTGTNGVPTDETVIVIRTPTTASTIITTTEPWNSTFTSTSTELTTVTGTNGVRT
DETIIVIRTPTTATTAITTTEPWNSTFTSTSTELTTVTGTNGLPTDETIIVIRTPTTATTAMTTTQPWND
TFTSTSTELTTVTGTNGLPTDETIIVIRTPTTATTAMTTTQPWNDTFTSTSTELTTVTGTNGLPTDETII
VIRTPTTATTAMTTTQPWNDTFTSTSTEITTVTGTNGLPTDETIIVIRTPTTATTAMTTPQPWNDTFTST
STEMTTVTGTNGLPTDETIIVIRTPTTATTAITTTEPWNSTFTSTSTEMTTVTGTNGLPTDETIIVIRTP
TTATTAITTTQPWNDTFTSTSTEMTTVTGTNGLPTDETIIVIRTPTTATTAMTTTQPWNDTFTSTSTEIT
TVTGTTGLPTDETIIVIRTPTTATTAMTTTQPWNDTFTSTSTEMTTVTGTNGVPTDETVIVIRTPTSEGL
ISTTTEPWTGTFTSTSTEMTTVTGTNGQPTDETVIVIRTPTSEGLVTTTTEPWTGTFTSTSTEMTTITGT
NGVPTDETVIVIRTPTSEGLISTTTEPWTGTFTSTSTEMTTITGTNGQPTDETVIVIRTPTSEGLISTTT
EPWTGTFTSTSTEMTHVTGTNGVPTDETVIVIRTPTSEGLISTTTEPWTGTFTSTSTEVTTITGTNGQPT
DETVIVIRTPTSEGLISTTTEPWTGTFTSTSTEMTTVTGTNGQPTDETVIVIRTPTSEGLVTTTTEPWTG
TFTSTSTEMSTVTGTNGLPTDETVIVVKTPTTAISSSLSSSSSGQITSSITSSRPIITPFYPSNGTSVIS
SSVISSSVTSSLFTSSPVISSSVISSSTTTSTSIFSESSKSSVIPTSSSTSGSSESETSSAGSVSSSSFI
SSESSKSPTYSSSSLPLVTSATTSQETASSLPPATTTKTSEQTTLVTVTSCESHVCTESISPAIVSTATV
TVSGVTTEYTTWCPISTTETTKQTKGTTEQTTETTKQTTVVTISSCESDVCSKTASPAIVSTSTATINGV
TTEYTTWCPISTTESRQQTTLVTVTSCESGVCSETASPAIVSTATATVNDVVTVYPTWRPQTANEESVSS
KMNSATGETTTNTLAAETTTNTVAAETITNTGAAETKTVVTSSLSRSNHAETQTASATDVIGHSSSVVSV
SETGNTKSLTSSGLSTMSQQPRSTPASSMVGYSTASLEISTYA*GSANSLLAGSGLSVFIASLLLAII*

Pezizomycota:

>KEY82091.1 hydrophobin [Aspergillus fumigatus var. RP-2014]
*MLVTMRLSRSIAVFTLVTYATG*LPSLQVIPRGEPQLPLLDPSMTIKEAARKCGDKAQLSCCNRVVKAGDY
TSVDEGIGAGLLSNLAGGGSGISSILAFDQCSRLDAQVPVVLLPIQDLLNQHCKQNVACCQKNPGDASS*S*
GVGVSLPCIALGSVA
[No TANGO seqs]

>EAL91055.1 conidial hydrophobin RodB [Aspergillus fumigatus Af293]
*MKFLAVVSLLAATALA*LPNAGVVHPTFASADKYTLQQAQNKCGEHTTLSCCNHVSKVGDTTAFNYGLLNG
LLGNAISGPEGVGILSGCQKISVTALIGVDDLLNKQCQQNVACCQDNKSVATGGLINIATPACVALDSII

>EAL91643.1 conidial hydrophobin Hyp1/RodA [Aspergillus fumigatus Af293]
*MKFSLSAAVLAFAVSVAA*LPQHDVNAAGNGVGNKGNANVRFPVPDDITVKQATEKCGDQAQLSCCNKATY
AGDVTDIDEGILAGTLKNLIGGGSGTEGLGLFNQCSKLDLQIPIIGIPIQDLVNQKCKQNIACCQNSP*SD*
ASGSLIGLGLPCIALGSIL
[No TANGO seqs]

>KDQ22759.1 vegetative mycelium hydrophobin 2 expressed in monokaryotic and dikaryotic micelia [Pleurotus ostreatus PC15]
*MFSRVMFCTFLILPLLAAATA*IPRTDTPSCSTGSLQCCSSVQKASDPLVGIIVALLGIVLGPLDLNVGLT
CSPITVIGVGGTSCTQQTV*CCTGNNFDGLIVAGCSPINIGL*

>EGX49926.1 hypothetical protein AOL_s00076g567 [Arthrobotrys oligospora ATCC 24927] (MAD1)
*MKGAIQFLGALAAVQAVSA*TYIDWTQPFNSYDCGGKQCGGRPKFEPPAYSNERCTPQQNTGYDFSDAPDG
DLPKYDDFDFSGYKCQKSKLQRRSGRGSGSKCASSYVEPETYSNEIKCGKKFSVDEFDISLEYESVIEFH
YGMPDGSSCKHVSKCGTGITPVKNTQCGGAKSVKCKIHKSSQNKKKCKFNIHHIKFRCDKPSTTSAPVPA
TTSEPAPCTEYSCTATDTTTEPAPTEPAPTEPAPCTEYSCTATDTTTEPAPTEPAPCTEYSCTATDTTTE
PAPCTEYSCTATDTTTEPAPTEPAPTEPAPCTEYSCTATDTTTEPAPTEPAPTEPAPCTEYSCTATDTTT
EPAPTEPAPCTEYSCTATETTSEAVPTTTDEAPCTDYSCTATEAVPTTTDEAPCTEYSCTGVPTSEAVPT
TSDDVPTTTDVYIPPTDVYVPPTDIYVPPANTSIPYETPSPSETETLPPSGTDVYTTLPSVPVETGCPPV
LPQCMETWTKITQCVNSGDVKCLCPNPEYIKSVAECVEAWGVDDDEVAKALEYMQGLCAEHIPENPAIVT
CVPTYVTLPPVTTGASTVTVSTTVVVPVTTASPEETNKPGYVPVFTTETVIRTVTVCPVKLVTTEPSKPV
LVPGTITAPPYVPPTAPATIPATVPAEATTPPVEYAPSTLMTAYPTVPVPVNNTTPNPPIATG*AASSFKA*
FSTVMLAGVIGLTALIMA

>AAA91036.1 WI-1 adhesin [Blastomyces dermatitidis]. (Bad1)
*MPDIKSVSSILLLVSSSLVAA*HPGARYPRDDKYPVNVKYSEHFHHPKCDWHLWDQWCNGDGHKHFYDCGW
GLTHPNYNYRLWKYWCDTKVHYNCELDESHLKYDAGLFKSLCTGPGKHLYDCDWPTSHVSYSWYLHDYLC
GNGHHPYDCELDSSHEDYSWPLWFKWCSGHGRHFYDCKWDNDHEKYDWPLWQYWCGSHDKDPYNCDWDKF
HEKYDWELWNKWCKDPYNCEWDSSHEKYDWELWNKWCKDPYNCEWNSFHEKYDWELWNKWCKDSYNCEWD
SSHEKYDWELWNKWCKDPYNCDWDSSHEKFDWGLWSHWCNDYDKYPYNCEWDSSHKKYDLTLWNRWCSSY
DKDPYKCDWDLWNQLCSGNGHHFYDCDWDVSYPGYDSHLWDLLCTNNPYNCEWDSSHEKYDWELWDKWCK
DPYNCDWDSSHEKYDWDLWNKWCKDPYNCEWDSSHEKYDWELWDKWCKDPYNCDWDSSHEKYDWDLWNKW
CKDPYNCEWDSSHEKYDWELWDKWCKDPYNCEWDSSHEKYDWKLWDKWCKDFYNCEWDSSHEKYDWELWD
KWCKDSYNCDWDKFHEKYDWELWDKWCKDSYNCDWDKFHEKYDWDLWNKWCKDSYNCDWDKFHEKYDWEL
WDKWCKDSYNCDWDKFHEKYDWELWDKWCKDFYNCEWDSSHEKYDWELWDKWCKDPYNCEWDSSHEKYDW
ELWDKWCKDFYNCDWDKFHEKYDWVLWNKWCKDPYNCEWDSSHEKYDWELWDKWCKDPYNCDWDKFHEKY
DWDLWNKWCKDPYNCEWDSSHEKYDWELWDKWCKDPYNCEWDSSHEKYDWKLWDKWCKDFYNCEWDSSHE
KYDWELWDKWCKDPYNCEWDSSHEKYDWELWDKWCKDPYNCEWDSSHEKYDWELWNKWCKDPYNCEWDSS
HEKYDWELWDKWCKDFYNCEWDSSHEKYDWELWDKWCKDPYNCEWDSSHEKYDWELWDKWCKDFYNCEWD
SSHEKYDWKLWNKWCKDFYNCEWDSSHEKYDWKLWNKWCKDFYNCEWDSSHEKYDWELWNKWCNKHDEHD
KHPWCPVCDPLSGANRCHPTTSCIGTGHSYYCACRAGYKSSHYSHDHKNFRLPFPGYEFLVFTPPGTECD
VLCDGYPHKPAHKLCSEVKVHNYCEP

Taphiniromycota

>AAR00228.1 INT1 [Pneumocystis carinii] no sig
MSSKTVEKHENLELDLKSGEKVAYEESERANSPLSAFFYEVNRIRRGSDRKKRRDGNTGSYGLLISSYNL
SKRDLSDIKSAAFFNRRYGVNCSTESLREGSGVYMRGDGMFSRTTVDSGQIFDESSSVKTSLDYDIYDGN
NVYKVIDSPLKAPTIHNVEYREYYDKNLCIKESNNEVNIFENEVSKVPEKKSFFLKGARKSSSGKGSEKK
STLRSISLNLKNLITSIVSNNDSHANQAQIENKDEGKSLQKENEFSDPTVLKTENVLGGNLSNEKRDMTK
SSFSEFYSRNLRKDIQSVEENEAIKMKEDVPKRFLEDEEKNKNKDVTVNVFKNSKNDYHMSKKNYQTYVS
NDDQEIFNDDLKYKYNSEDVPYDDGIKNNAEKLKTNIEESSVYESDSEISQRLPSYCKLDAIDIGNFELN
DFMIRKSSNISTKEISQFKSKIFEDEALNEKKINDSNTEQEEKSYIKSNDFSDKLTKADSNFEISHSDAE
KYKPDNQPLNDNKNANIYQSTVNGDSNISISLPLLGDQLLSDFNVSLDDVSDAIDKVIEVRKVCIKLFNR
NVFNCLAWLYYETECKYSHASSNDVCEKDTKRERYASPKIDDIYEKNEKHPVLKFDKTKNIVINKVPQDD
SIGKKPDSGLLFIRVIEIENLDLPLPEDKTLRFCCTLDNGRHYITTPWIPFTKNAKINEEFELVANDDLE
FTLTLRLDYQPSVDHKKKDFIFSRIFSSSKKQGALSSFNPPNCCLSNNGSFGRSYLSLKMFKDHAFGCPY
TTTVSCMNEWTEKAVVKGRKRQILKVKSYIICQLKINAFYVPFVPGQAKETMPKSLSACVKDIKNAEWVS
KLHYEGFLIQHGGDCLYRKRRYFRLSGLKLTSYHESSKSIRSNINLAKVKTILNDQQLSIDSKKMTRAVH
TENNKQSSTLEGDNDYVLTQNGFCIKFFNGEIINFYADSEDEKARWVRVLDAVIKKANQVKPWCLYVLQK
QKAMKRFPPKSALKKKKLSFLIYSN

>AAA33797.1 major surface glycoprotein [Pneumocystis carinii] no sig
?MARPVKRQAVQGAQDEIDEKHLLAFIVKDKYKEEQKCKEELEKYCKELKEADKNLENVDDKVKGLCDDKK
RDEKCKDVKKKVEDELKDFEEELQKVLNNIKDENCEKYEEKCILLEETDYDVIKDNCIELREGCYKLKRE
KVAEELLLRALGGDAKEEAKCKGKMNTVCPVLSRESDELMSFCLDSAKTCGDLKKKLGTVCEPLKKELKD
NELAEKCHERLEKCHFYGEACDDAKCKKFEEQCKGKNIIYKAPESDLSPVKPRASLLRSIGLDDVYKNAE
KHGIIIGKSGVDLPRKSGTKFLQDLLLLLSRDEDKKEPDKKCTKALEKCDASKYLNTELEKLCKDGNKNE
KCKKILDVKERCTNLKLKLYLKGLSTEYDDQESDPLSWGQLPTFFIKGECAELESECFYLEKACKDNNID
KACQNARAACYKKGQDRMLNKFFQKELKGKLGHVRFYSDPKDCKKYVVENCTKLDKKYLPRCLYPKELCY
GLSNDIFLQSKELSALLDDQRDFPLKKDCVELKEKCDELSSDSLLNLEKCITLKRRCEYFRVSEGFRNVF
LEKKDDSLMTQDNCTKALHEKCHQLYRRRKNSFSVSCALPEETCSYMVFHTSQDCSSLKVNIKNEKILEK
IGEEIKKANKNEALVEELCTTWGRHCHQLMENCPDDLKKKENGNGNDHNCEALQEKCNKTFEKLKLEEEL
SHLLKGSLKDDKCKEALGKRCTELEKNEAFKTLYGKCDDNTKENVCKKLVDKVKKRCPTLKDELENAKKE
LTKMKNEYDDLKKAAEKSTEAAKLLLSRPRQTVMPNAQNGSDSTLVPPPPQAPAGPPPPGSPPPPPSQNG
TPGTPGGETGASGGTPGTPGTPGTPGTPGGMMKYAKLGLVKRTYVDGGVSEVEVKAFDATTIALELYLEL
KEECKALELDCGFKEDCPDTKQACENIDTLCKLEPLEIKPHHTEKITETKTETKTETKTETKTDGKADEK
TVEKTVETKSVGGGKVTEECTMIQTTDTWVTSTSLHTSTTTSTSTVTSTVTLTSMRKCKPTKCTTDSSK
ETQKEEDDEEVKPNEGMK*IRVPDMIKIMLLGVIVMGMM*

>sp|Q9P6S0.3|GSF2_SCHPO RecName: Full=Galactose-specific cell agglutination protein gsf2; AltName: Full=Galactose-specific flocculin; AltName: Full=Pombe flocculin 1; Flags: Precursor
*MSVRRFLSTSARALLFTAALLPSLTSGLPSG*NVRILQKGMEPEDYLSSASQNEVPHDISLPKTELADPNF
LVDDMPTLLGRDAAVDPSMFTSTFTVKNGNDANYITASPVSNDASMTAISTFTSGKEASYAIQASPSTFL
PDSTTTSGSQVSNAVEASSTFVADTTSTSCNPATVLIVTTSGSTSTSCPPPTTILIVTVPTTTTTTTVGY
PGSVTTTLTGTPSNGTVIDTVEVPTTTNYGYTTITTGYTGSTTLTTTVPHSGNETGPTTVYVETPYPTTV
TTTTTVGYPGSVTTTLTGAPSNGTVIDTVEVPTTTNYGYTTVTTGYTGSTTLTTTVPHSGNETGPTTVYV
ETPYPTTVTTTTTVGYPGSVTTTLTGAPSNGTVIDTVEVPTTTNYGYTTVTTGYTGSTTLTTTVPHSGNE
TGPTTVYVETPYPTTVTTTTTVGYPGSVTTTLTGAPSNGTVIDTVEIPTTTNYGYTTITTGYTGSTTLTT
TVPHSGNETGPTTVYVETPYPTTVTTTTTVGYPGSVTTTLTGAPSNGTVIDTVEVPTTTNYGYTTITTGY
TGSTTLTTTVPHSGNETGPTTVYVETPYPTTVTTTTTVGYPGSVTTTLTGAPSNGTVIDTVEVPTTTNYG
YTTVTTGYTGSTTLTTTVPHSGNETGPTTVYVETPYPTTVTTTTTVGYSGSVTTTLTGSGSNSIVTETVD
VPTTTSVNYGYTTITTGWTGSTTLTSIVTHSGSETGPTTVYIETPSVSATTTTTTIGYSGSLTTTLTGSS
GPVVTNTVEIPYGNSSYIIPTTIVTGTVTTVTTGYTGTETSTVTVIPTGTTGTTTVVIQTPTTVTATETD
IVTVTTGYTGTETSTVTVTPTGTSTGTTTVVIQTPTTVTATETDIVTVTTGYTGTETSTVTVTPTGTSTG
TTTVVIQTPTTVTATETDIVTVTTGYTGTETSTVTVTPTGTSTGTTTVVIQTPTTVTATETDIVTVTTGY
TGTETSTVTVTPTGTSTGTTTVVIQTPTTVTATETDIVTVTTGYTGTETSTVTVTPTGTSTGTTTVVIQT
PTTVTATETDIVTVTTGYTGTETSTVTVTPTGTSTGTTTVVIQTPTTVTATETDIVTVTTGYTGTETSTV
TVTPTGTATGTTTVVINTPTTTGSEVLPTTGATGTAGTETQLTTATEVQPTTGATGTAGTETQVTTGTET

QATTATETQATTATEVQTTTGATGTAGTETQATTATEVQPTTGATGTAGTETQVTTATEVQPTTGATGTA
GTETQVTTGTETQATTATETQATTATEVQTTTGATGTAGTETQATTATEVQPTTGATGTAGTETQVTTAT
EVQPTTGATGTAGTETQVTTGTETQATTATETQATTATEVQTTTGATGTAGTETQVTTATEVQPTTAVTE
TSSSGYYTTIVSSTVVSTVVPGSTVYPVTHVTTTTGVSGESSAFTYTTSSTQYEPSTVVTTSYYTTSVYT
SAPATETVSSTEAPESSTVTSNPIYQGSGTSTWSTVRQWNGSATYNYTYYTTGGFTGGNNTNVTGLYP*SS*
AGANKPIAYLTFVSLFVYIVTLI

>CAC19750.2 cell agglutination protein Mam3 [Schizosaccharomyces pombe]
*MSIALAFFILVLLGFSWA*SPSALDDTFNVSRSVGLISSSNLESCQSSPLEVGNIYNSTSASEILSTLDAK
YITIIGVIGSSNSSIQDLIDSVGNSNNAASSNPTSTVTEYVDRVQTVTEYVTLSCGQAFTSTVDISSSTS
SSVINSPTGTAVSSQISTLSMSPSSTPVFSPSASVSSKVASSVSYVSSEPSDSSSSTNTVILTTSVNSPA
VSSSETLTSVSITSTESAYTSSSVDIAASTTASSTLPVSTSEATVSFSTDIPATPSTLSSPASSSSSYLV
ETSSTLTDSVFTTVTATSDSSVITYTLINSVTSSSETTNLPSSSSSLVTIGESSFPSSLLSLLTQSFSTV
RSTSSSSTDQLTSASPISSSVISPSVSSPTSSILTNSGSIKSGDHQIVTTSFVQTTTHGSQVETLTYVTT
LTETILTTTYDSHTFLTTITPSPSNSISYTNNTFIPSSSIKSSIVYSVTPTSAENYTSSEAFSTSSSLVV
IPPVNSSLVTSSTSFTKFSSLSSSQLSTENFTSASSSLSLTNAKSSLSTPSTTIPTSNSSVSLQTSSSLI
ISSPIISSSLTATSTSTPALTHSITPSNTSYTSSLIPSSSTDYSSSLITVCSNVTSEISSTSLASLISTL
TSQQISSNKSSEFVGQTTTEYTTSGSVGFTTTLATQSGSVPGTVLVDVPTPSWITETVTSGSVGFTTTIA
TPIGTTAGTVLVDIPTPSWVTETVTSGSIGFTTTIATPIGSTAGTVLVDVPTPSWVTETVTSGSVGFTTT
IATPIGSTAGTVLVDIPTPSWVTETVTSGSVGFTTTIATPVGTTAGTVLVDIPTPSWVTETVTSGSVGFT
TTIATPVGTTAGTVVVDVPTPSWVTETVTSGSVGFTTTIATPIGSTAGTVLVDIPTPSWVTETVTSGSVG
FTTTIATPVGTTAGTVLVDIPTPSWVTETVTSGSVGFTTTIATPIGTTAGTVLVDIPTPSWVTETVTSGS
VGFTTTIATPVGTTAGTVLVDIPTPSWVTETVTSGSVGFTTTIATPIGTTAGTVLVDIPQQHATTTTTTT
FDGFSGYTSSYTGSITETIVIGTPHHSVVDVS

>NP_596007.1 cell surface agglutination protein Map4 [Schizosaccharomyces pombe]
*MNSYAILLSLFFSFERLLTLANA*NSLYSPFNNSSFVDSDTSFSDLSRNGLLSLLDSNTTSASVQTIAISQ
TDNAASCIPSASLLSSSVVLYSAKETVTVSSYWSLVSTSVTGTVYVPYTSSVACFPYATSDAPNPIPRGD
SATSTSIAPTYSASDSSATTITSSSPSTSIIGTGSTDTSVSSTLTYHTPIASPTTSSNSDNEYTVDVITS
SSLSSFVITNVDSTTTSVINYIGASTLESSSLTNTVSPTESTFYETKSSTSSVPTQTIDSSSFTSSTPVS
LTSSSTSSSGSSQDSTTIDSTPSTIATSTLQPTTSSPITTSAPSLSSALPTTYPSSLSTEVEVEYFTKTI
TDTSSIVTYSTGVETLYETETITSSEISSIIYNFSTPISGSSFPDGFKPINPTSFPSLTSSTKKIPSTTL
PTSSKMITTTTPSVSNNTQSSFLIISTFTSSYEHSEPFKVSSVPLTSNNFSSISHSSASSLPITPSSYLS
NTTLHSSVQSSQSSQFTVSVPSSTQSYSTSSNFTTPITISTSLSSFPTTIVSSSFQYSSLSSNVTTTNAQ
SSSLSSSNSSALTHISSSIVSSGSSSALSSSTIVSSINSSSSVFISSVSSSLQYSSSYVTETTTSGSVGF
TTTIATPVGSTAGTVVVDIPTPSWVTETVTSGSVGFTTTIATPVGSTAGTVLVDIPTPSWVTETVTSGSV
EFTTTIATPVGTTAGTVVVDIPTPSWVTETVTSGSVGFTTTIATPIGTTAGTVLVDIPTPSWVTETVTSG
SVGFTTTIATPVGTTAGTVLIDVPTPTASSSPFPSCNTQCTNENSFRIQVINDDIYPSYVHLDSNNYAIA
AARGDSDGENVFIYDSDIKRIVSCCGVKPIYRLDQDDTEGYSFEIYKDNDGQLQFKYPLNDALYPMELLT
LTDGRIGITTNLTLYKPYYLNNVENERAANVVLRALEY

Basidiomycete:

>XP_012046965.1 hypothetical protein CNAG_00795 [Cryptococcus neoformans var. grubii H99] (Cfl1)
*MLVSNIFALLGLASYAHA*NGISLAMGTRINVGVSTSITAKASVSERNSCSSPGDFFTQLLDNPLCSSPHT
RTTPSSDLTCPFNWFMHKTAKCCIPQTEVAPCDCGEGYTFNEKTKKCVKNAGSCSGHQWWHQRSSSCCDN
SWHTSPPKGSCPSGITCPTNWFWHKTLKKCKPLHPRAPEPDCDNWDSHKQCCGSGGSSPSQTASKGKGKR
TLQARQQQTLYPQTELDRMYCPGSLHACTVNSGSGGEWAYECVDFATELESCGGCSSTGEGQDCTQIPNA
FSVGCELGSCAVYSCKSGFRLNGTE**CIAV**

> AJ938050 1233 bp mRNA linear PLN 24-OCT-2005 Filobasidiella neoformans mRNA for deacetylase (MP84 gene)
*MYGHLSLSTLSLLAVVAA*APFHESWLQPRDSDVSQLFRRGAPDPKASDYLSYYPSPGSTPNVSTIPQAWLDKLATVQ
LPNVSVATANDGRPTYPNNENDGDSEICSFTDQCYVEDDLYSPPGEKVWALSFDDGPTDVSPALYDYLAQNNISSSA
THFMIGGNIITSPQSVLIAIEAGGHLAVHTWSHPYMTTLTNEQVVAELGWTMQALSDLNGGRIPLYWRPPYGDVDNR
VRAIAKGVFGLVTVLWDSDTNDWAISDQPDQYSVASVEAYFDTLVTGNRTQGLLLLEHELDNNTVEVFETEYPKAVA
NGWSVKNVADAFSMKWYLNSGKGNDDVVTTMSVAGTLTTAKPTHTSTSVASATATSSASVTDSAGVSIAS*AASSQES*
SSWPIANSLFVIACGLALAAIMV
